# Intracellular oxygen metabolism during bovine oocyte and preimplantation embryo development

**DOI:** 10.1038/s41598-021-99512-5

**Published:** 2021-10-28

**Authors:** Paul J. McKeegan, Selina F. Boardman, Amy A. Wanless, Grace Boyd, Laura J. Warwick, Jianping Lu, Keerthi Gnanaprabha, Helen M. Picton

**Affiliations:** 1 Reproduction and Early Development Research Group, Leeds Institute of Cardiovascular and Metabolic Medicine, School of Medicine, University of Leeds, Clarendon Way, Leeds, LS2 9JT UK; 2 Centre for Anatomical and Human Sciences, Hull York Medical School, University of Hull, Hull, HU6 7RX UK; 3 CARE Fertility, Manchester, England UK; 4 Assisted Conception Unit, Ninewells Hospital, Dundee, Scotland UK; 5 Department of Biological Sciences, University of York, Wentworth Way, York, YO10 5DD England UK; 6 St James′s University Hospital, Beckett Street, Leeds, LS9 7TF England UK; 7 GCRM Fertility, 21 Fifty Pitches Way, Glasgow, G51 4FD Scotland UK

**Keywords:** Embryology, Cellular imaging, Energy metabolism

## Abstract

We report a novel method to profile intrcellular oxygen concentration (icO_2_) during in vitro mammalian oocyte and preimplantation embryo development using a commercially available multimodal phosphorescent nanosensor (MM2). Abattoir-derived bovine oocytes and embryos were incubated with MM2 in vitro. A series of inhibitors were applied during live-cell multiphoton imaging to record changes in icO_2_ associated with mitochondrial processes. The uncoupler carbonyl cyanide-p-trifluoromethoxyphenylhydrazone (FCCP) uncouples mitochondrial oxygen consumption to its maximum, while antimycin inhibits complex III to ablate mitochondrial oxygen consumption. Increasing oxygen consumption was expected to reduce icO_2_ and decreasing oxygen consumption to increase icO_2_. Use of these inhibitors quantifies how much oxygen is consumed at basal in comparison to the upper and lower limits of mitochondrial function. icO_2_ measurements were compared to mitochondrial DNA copy number analysed by qPCR. Antimycin treatment increased icO_2_ for all stages tested, suggesting significant mitochondrial oxygen consumption at basal. icO_2_ of oocytes and preimplantation embryos were unaffected by FCCP treatment. Inner cell mass icO_2_ was lower than trophectoderm, perhaps reflecting limitations of diffusion. Mitochondrial DNA copy numbers were similar between stages in the range 0.9–4 × 10^6^ copies and did not correlate with icO_2_. These results validate the MM2 probe as a sensitive, non-toxic probe of intracellular oxygen concentration in mammalian oocytes and preimplantation embryos.

## Introduction

Regulated mitochondrial function is responsible for energy-releasing metabolic reactions, cell signalling and apoptosis and is vital to the developing embryo. Mitochondrial dysfunction may lead to developmental arrest, embryo death or developmental failure *in utero*^1–3^, and has been implicated in the development of a range of diseases in childhood or adulthood, such as autism^4,5^ and Alzheimer’s^6–8^. Furthermore, studies suggest that mitochondrial DNA content may predict embryo viability in assisted reproduction therapies^9^. More detailed understanding of mitochondrial function in oocytes and developing embryos is however needed to improve our understanding of how early development affects health and disease throughout life.

### Oxidative phsophorylation

The primary function of mitochondria is to provide energy in the form of Adenosine Triphosphate (ATP) which is coupled to the consumption of oxygen by oxidative phosphorylation (OXPHOS)^10^. Therefore, ATP synthesis and oxygen consumption are tightly linked and regulated. Whilst glycolytic activity also produces ATP in oocytes and early embryos, dramatically more ATP is produced by OXPHOS^11–13^. Oxygen consumption therefore directly correlates to levels of aerobic respiration and is used as a marker of overall metabolism in mammalian oocytes and embryos^12,14^ which may reflect embryo viability^15^. OXPHOS reportedly increases during preimplantation embryo development to provide an increasing proportion of total ATP in order to meet the increasing energy demand of protein synthesis and blastocoel formation^15,16^. Additionally, by the blastocyst stage, cells have differentiated into trophectoderm (TE), from which placenta tissue is derived, and inner cell mass (ICM), which gives rise to the fetus. These cell types are believed to have different metabolic phenotypes^17^, with TE notably more metabolically active than ICM. However, there is currently no established method to measure TE vs ICM metabolism without biopsy.

### Measuring oxygen metabolism

Due to the vital role of oxygen in mitochondrial metabolism, several methods of measuring oxygen consumption in the oocyte and embryo have been reported. Contemporary and emerging methods include the Unisense Nanorespirometer^15,18^, the self-referencing electrode^19^ and bespoke electrochemical devices^20,21^. Recent developments include combining electrochemistry with microfluidic technologies^22,23^ for embryo-compatible devices. Obeidat and colleagues^24^ report detecting oxygen consumption alongside glucose consumption, lactate production and pH in individual equine blastocysts using multiple electrochemical sensors. Extracellular flux analysis using the Seahorse Bioanalyzer (Agilent) has recently been published as a more user-friendly, rapid method of profiling the components of respiration in small groups of oocytes or preimplantation embryos^25^. However there has to date been no report of changes in intracellular oxygen concentration (icO_2_) within the oocyte or embryo itself.

### Probing mitochondrial function

As noted previously, coupling of mitochondrial oxygen consumption to ATP production is tightly regulated within tissues. However, it is notable that tissues can modulate mitochondrial oxygen consumption and ATP production to fulfil other roles, such as heat generation by brown adipose tissue^27,28^. And that the contributions of different mitochondrial complexes and processes can be elucidated using metabolic poisons, as recently reported by the Sturmey group^25,29^. Exogenous uncouplers, such as carbonyl cyanide-p-trifluoromethoxyphenylhydrazone (FCCP), disrupt the regulation of the proton gradient and increase mitochondrial oxygen consumption to its maximum, whilst preventing production of ATP by OXPHOS^14,25,28–30^. FCCP also leads to reduced ATP content in human oocytes^31^, as well as increased glucose consumption and a shift in redox state in equine embryos^31^. The difference between basal and maximal oxygen consumption reveals the spare respiratory capacity; a phenomenon which can enable tissues to adapt to changing ATP demand^30^. Inhibitors of mitochondrial protein complex III, such as antimycin, block mitochondrial oxygen consumption and mitochondrial ATP production completely^14,25,28–30^. These are used to quantify the contributions of mitochondrial and non-mitochondrial oxygen consumption in vitro.

### Embryo mitochondria

It is an essential requirement that the number, localisation and function of mitochondria are sufficient to support oocyte developmental competence through OXPHOS^3,26,32^. Indeed, oocyte maturation reportedly involves a burst of mitochondrial DNA replication, as indicated by mitochondrial DNA copy number^33,34^.

However, the number of mitochondria per embryo remain fixed from the 1 cell zygote to the 100–200 cell blastocyst, as the cellular machinery necessary to replicate the organelles are not present until postimplantation^26,36,37^. Mitochondria mature from a globular, condensed form into the elongated classical structure with an increased number of cristae during preimplantation development^26^. This occurs in parallel with an increase in OXPHOS activity from the zygote to the blastocyst^52,66,67^. It is tempting to speculate that the resulting increase in surface area:volume ratio could increase mitochondrial function without changing the number of mitochondria. This study therefore aims to investigate whether sensitivity of icO_2_ to maximal and ablated mitochondrial oxygen consumption, indicated by FCCP and antimycin respectively, change during in vitro oocyte maturation and preimplantation embryo development in line with mitochondrial maturation.

### The MM2 icO_2_ probe

One reported method to measure icO_2_ in mammalian cell culture is the multimodal MM2 probe, developed by Papkovsky and colleagues^38^ Several studies report that this and similar nanoparticle probes are suitable for highly sensitive ratiometric measurements in a variety of tissue types including monolayer and spheroid cultures^38–42^. MM2 is an intracellular label consisting of an oxygen-sensitive phosphorescent reporter dye Pt(II)-5,10,15,20-tetrakis-(2,3,4,5,6-pentafluorophenyl)-porphyrin (PtTFPP; emission at 650 nm) and a poly(9,9-dioctylfluorene) reference fluorophore (PFO, emission at 410–450 nm), within 70 nm nanoparticles^38^. The probe is ratiometric; the PtTFPP emission peak at 650 nm is quenched by oxygen, while the PFO emission peak at 440 nm is oxygen insensitive and hence used as a loading control. Quantification is possible using a simple 2-point calibration curve comprised of an aqueous solution in atmospheric conditions (21%, normoxic), and a second fixed sample with a chemical oxygen scavenger to remove all oxygen from the surrounding solution (0%, anoxic)^40^. Samples must be fixed to eliminate any effect of oxygen consuming activity. This 2-point calibration approach is widely used in the literature, including by the Papkovsky group, who developed the suite of probes including MM2 and corresponding protocols^40^, but also across a wide variety of fluorescence-^43–46^ and electrode-based^18,45–48^ methods to profile oxygen metabolism. To the authors’ knowledge this is the first example of the use of the MM2 probe in mammalian oocyte and embryo tissue.

**Figure 1:**
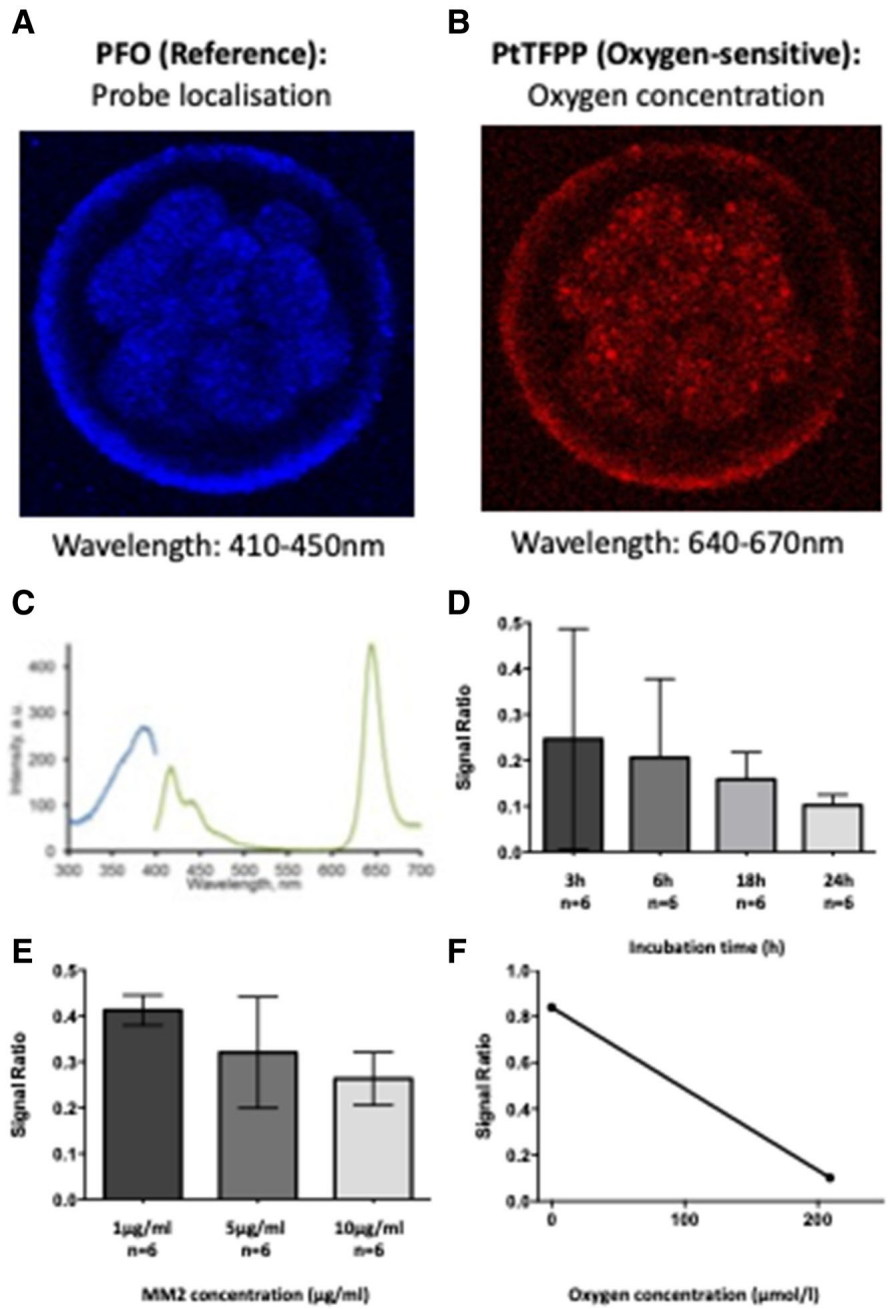
Optimisation and calibration of MM2 incubation conditions. Examples images (**A**) PFO oxygen-insensitive fluorophore labelling; (**B**) PtTFPP oxygen-sensitive labelling; (**C**) Excitation/Emission spectra reproduced from manufacturer guidance, (**D**) Signal ratio comparison following blastocyst incubation with MM2 probe for 3 (n = 6), 6 (n = 6), 18 (n = 6) and 24 h (n = 6), with no statistical differences; (**E**) Blastocysts imaged after 24 h incubation with 1 μg/ml (n = 6), 5 μg/ml (n = 6), or 10 μg/ml (n = 6) MM2 probe with no significant differences; (**F**) Example calibration based on blastocysts imaged at 0% and 21% O_2_ (p < 0.0001) with curve y = − 0.0035x + 0.84. Values plotted as mean ± SD for the number of analyses shown.

**Figure 2:**
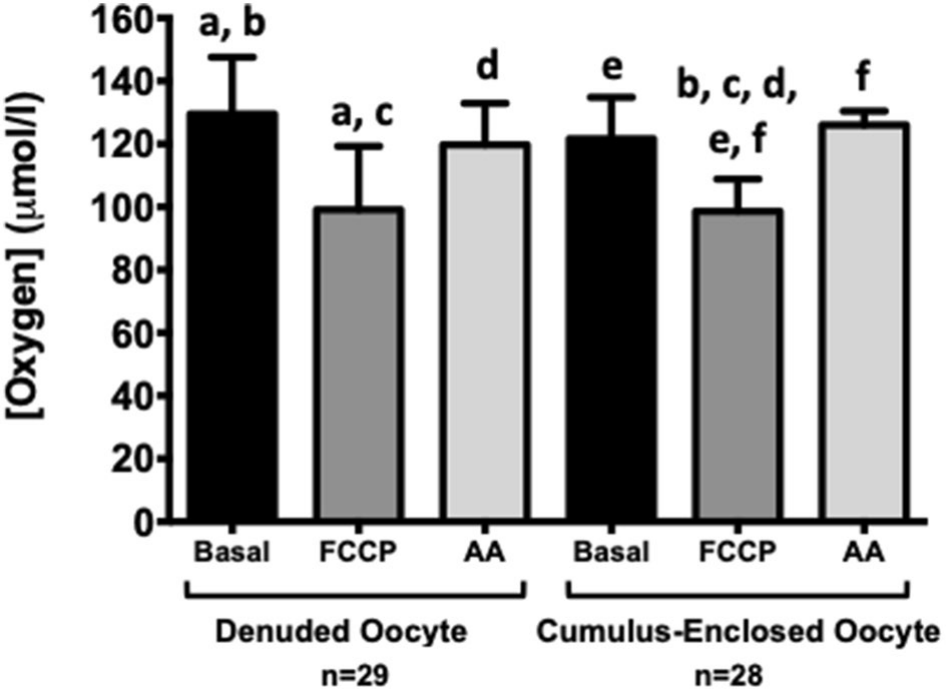
The effect of cumulus complex presence on oocyte oxygen concentration. Change in denuded oocyte icO_2_ (n = 29): Basal (129.50 ± 18.07 µmol/l), FCCP treated (99.13 ± 20.14 µmol/l, p = 0.0002) and antimycin treated (119.7 ± 13.20 µmol/l, p > 0.05). Change in cumulus-enclosed oocyte icO_2_ (n = 28): Basal (121.60 ± 13.15 µmol/l), FCCP treated (98.60 ± 10.20 µmol/l, p < 0.0001) and antimycin treated (126.00 ± 4.40 µmol/l, p < 0.005). Values plotted as mean ± SD for the number of analyses shown. Values with the same letter indicate significant differences by ANOVA with post-hoc Tukey test.

**Figure 3:**
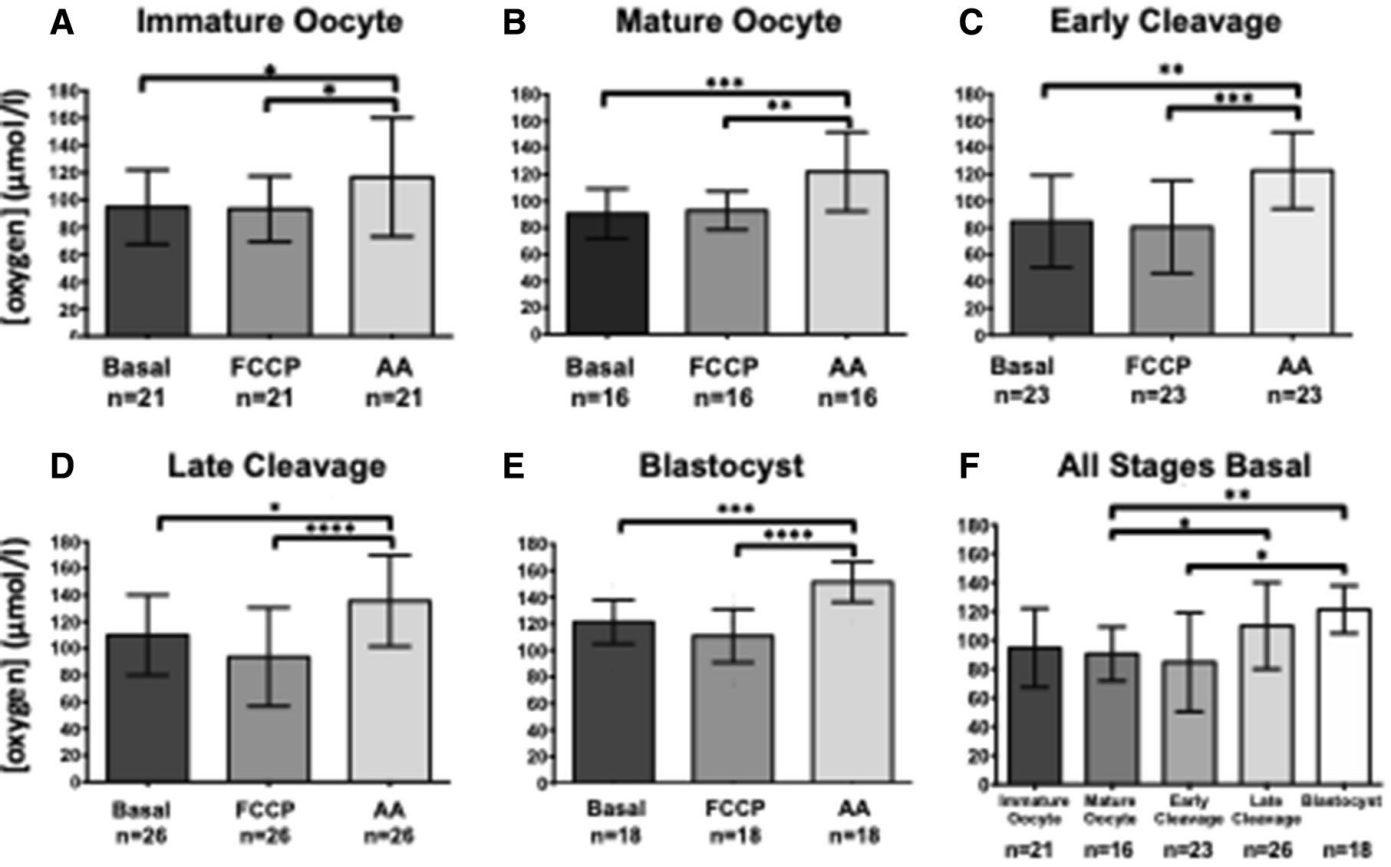
icO_2_ throughout preimplantation development. Effect of FCCP and antimycin treatment on icO_2_ of (**A**) immature oocytes (n = 21) Basal (94.76 ± 27.28 µmol/l), FCCP treated (93.39 ± 24.06 µmol/l) and antimycin treated (116.6 ± 43.59 µmol/l); (**B**) mature oocytes (n = 16) Basal (90.56 ± 18.73 µmol/l), FCCP treated (92.97 ± 14.46 µmol/l) and antimycin treated (122.1 ± 29.73 µmol/l); (**C**) EC embryos (n = 23) Basal (84.84 ± 34.37 µmol/l), FCCP treated (80.78 ± 34.66 µmol/l) and antimycin treated (122.90 ± 28.60 µmol/l); (**D**) LC embryos (n = 26) Basal (110.1 ± 30.03 µmol/l), FCCP treated (93.73 ± 36.91 µmol/l) and antimycin treated (135.70 ± 34.14 µmol/l); (**E**) Blastocysts (n = 18) Basal (121.4 ± 16.51 µmol/l), FCCP treated (110.0 ± 20.03 µmol/l) and antimycin treated (151.40 ± 15.16 µmol/l); (**F**) Basal icO_2_ comparison between immature oocyte, mature oocyte, EC embryo, LC embryo and blastocyst stages. Values plotted as mean ± SD for the number of analyses shown *p < 0.05, **p < 0.01, ***p < 0.001 and ****p < 0.0001.

**Figure 4:**
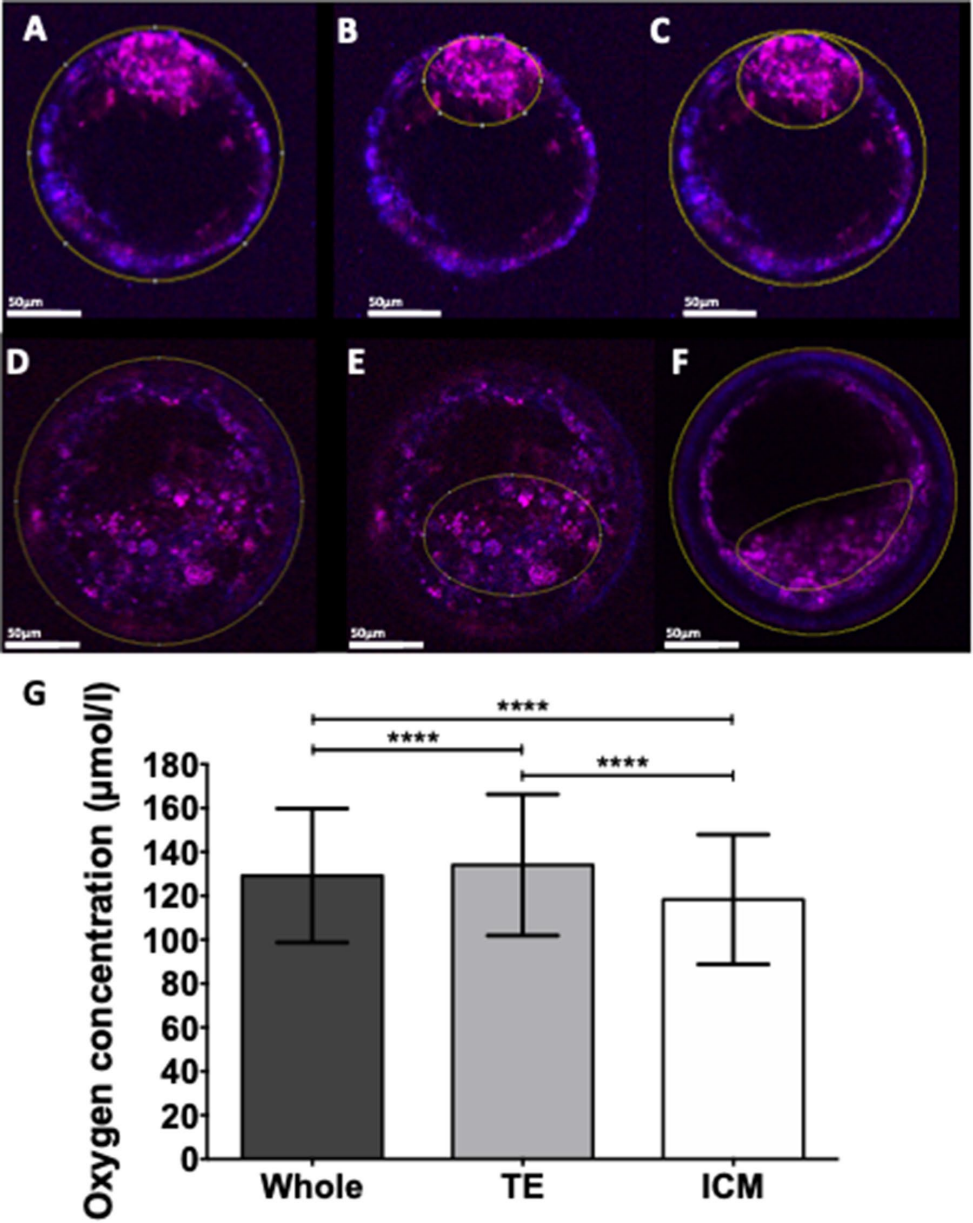
Blastocyst ICM and TE icO_2_. (**A**–**F**) Example of using ImageJ region of interest selection to measure relative signal intensity of (**A**, **D**) Whole blastocyst, (**B**, **E**) Inner Cell Mass (ICM) and (**C**, **F**) Trophectoderm (TE) regions. Merged images are shown; channels were measured individually. (**G**) Quantitative comparison of change in icO_2_ between whole blastocyst(129.23 ± 30.54 µmol/l), TE (134.10 ± 32.18 µmol/l) and ICM (118.33 ± 29.57 µmol/l, n = 82). Values plotted as mean ± SD for the number of analyses shown ****p < 0.0001.

**Figure 5:**
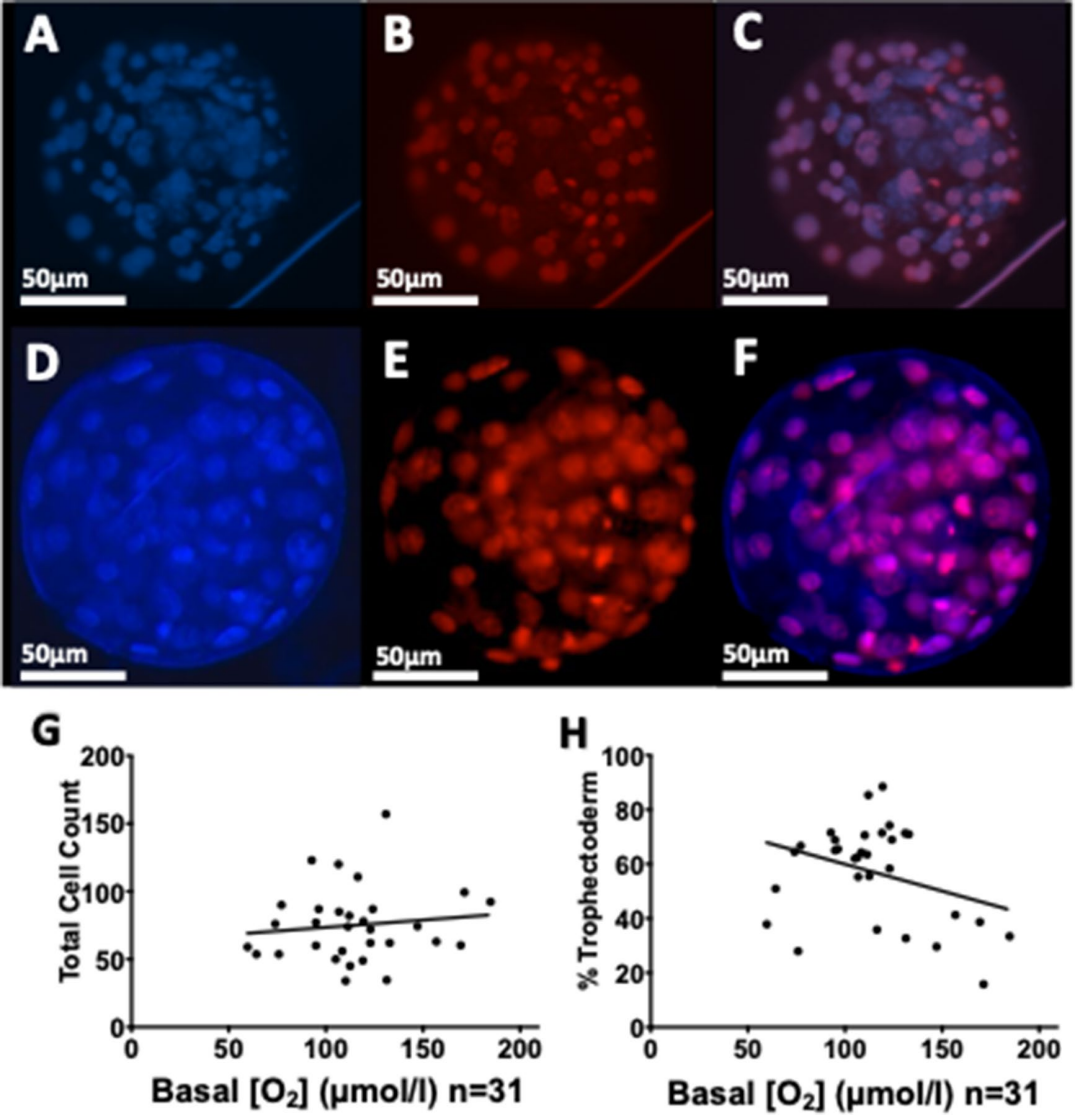
Blastocyst cell allocation and basal oxygen concentration. Examples shown of Hoechst total cell staining (**A**, **D**), Propidium Iodide (PI) Trophectoderm staining (**B**, **E**), combined channels (**C**, **F**). (**G**) Basal icO_2_ versus total cell count with line equation y = 0.1086x + 62.62, R^2^ = 0.015, p = 0.52. (**H**) Basal oxygen concentration versus % Trophectoderm with line equation y = 0.1974x + 79.68, R^2^ = 0.11, p = 0.07.

**Figure 6:**
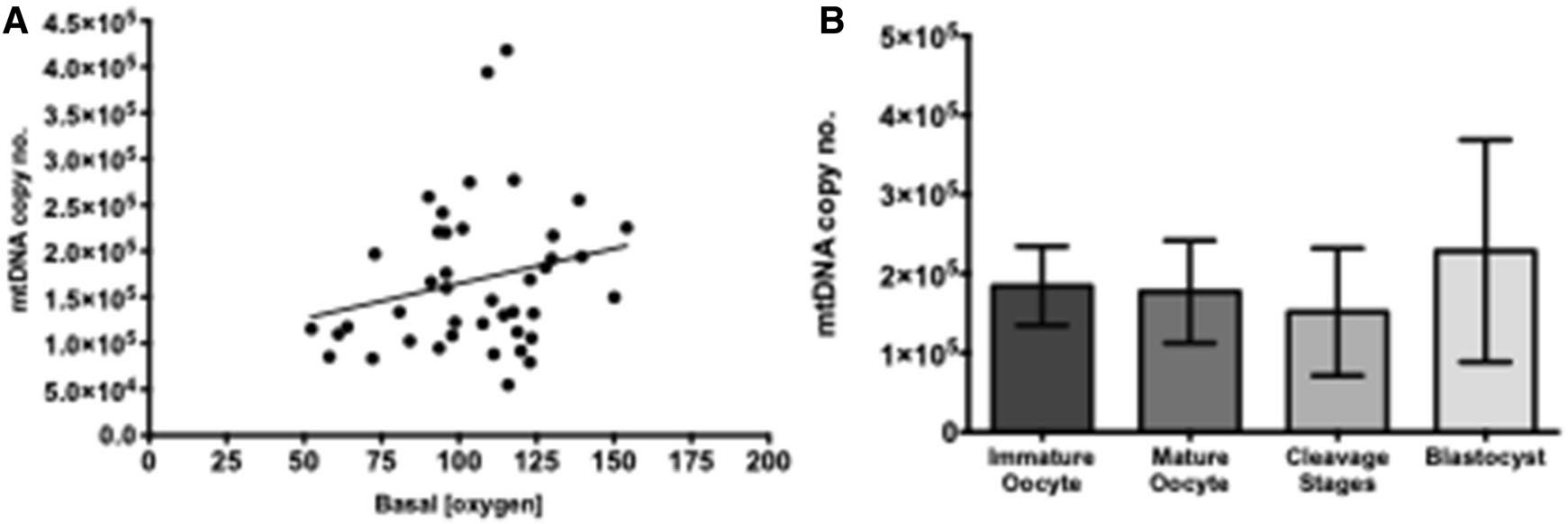
Mitochondrial DNA copy number. (**A**) Correlation of mtDNA copy number with basal oxygen level across all samples tested (n = 42) with line equation y = 759.7x + 88,713 (R^2^ = 0.05, p = 0.14); (**B**) mtDNA copy number for immature oocytes (1.75 × 10^5^ ± 1.6 × 10^4^ copies; n = 10), mature oocytes (1.77 × 10^5^ ± 2.6 × 10^4^ copies; n = 6), cleavage stage embryos (1.5 × 10^5^ ± 1.8 × 10^4^ copies; n = 19) and blastocysts (2.3 × 10^5^ ± 5.3 × 10^4^ copies), with no significant differences. Data shown as mean ± SD.

The aim of this work was to develop a new method to visualise icO_2_ in abattoir-derived individual oocytes and embryos in vitro, allowing resolution of different regions within the oocyte or embryo and identification of zones of high and low metabolic activity. We first optimised incubation conditions (Fig. 1) and verified whether cumulus cell layers were necessary for oocyte labelling (Fig. 2). We then used FCCP and antimycin to profile icO_2_ within the limits of mitochondrial control in oocytes and preimplantation embryos (Fig. 3). We then report use of the MM2 probe to quantify icO_2_ in ICM and TE regions of blastocysts (Fig. 4), correlation of icO_2_ to blastocyst cell count and cell allocation to ICM and TE lineages (Fig. 5) and finally correlation to mtDNA copy number (Fig. 6).

## Results

### Validation and optimisation of the MM2 probe in oocytes and embryos

Incubation times of between 3 and 24 h have been reported for loading the MM2 probe^38,49,50^. Groups of in vitro derived day 7 bovine blastocysts were incubated for 3, 6, 18 or 24 h with 10 µg/ml MM2 probe in SOFaaBSA before multiphoton imaging (n = 6 per group, total n = 24). The mean signal ratio did not differ statistically dependent on incubation time and ranged from 0.10 ± 0.01 IU to 0.25 ± 0.1 IU (p = 0.84, Fig. 1A). However, variation was lowest at the 24 h time point. Therefore, 24 h incubation time was selected for reproducibility and ease of use.

Next, blastocysts were labelled and imaged with 1 μg/ml, 5 μg/ml or 10 μg/ml MM2 probe in SOFaaBSA over 24 h (n = 6 per group, 3 independent groups). The mean signal ratio ranged from 0.24 ± 0.04 IU to 0.4 ± 0.02 IU (Fig. 1B). No significant difference in signal was found between the 3 different concentrations used (p = 0.8). An incubation concentration of 5 μg/ml MM2 was selected for all remaining experiments in line with loading concentrations previously reported by the Papkovsky group^42,50^.

2-Point calibration of MM2 signal with the corresponding sample of interest was performed in order to calculate icO_2_. An example blastocyst calibration curve is shown in (Fig. 1C). The mean signal ratio of fixed MM2-stained blastocysts under anoxia (0% O_2_) was 0.84 ± 0.002 (n = 3). This was significantly higher (p < 0.0001) than the mean signal ratio of fixed blastocysts labelled and imaged under normoxia (21% O_2_) (0.10 ± 0.04, n = 3), producing a calibration curve with the equation y = − 0.0035x + 0.84.

### The effect of cumulus cells on MM2 labelling efficacy and oxygen concentration

Oocytes were randomly assigned into denuded or intact cumulus-enclosed groups and labelled with 5 μg/ml MM2 for 3 h (Fig. 2). Denuded oocyte icO_2_ (129.50 ± 18.07 µmol/l, n = 29) reduced significantly following FCCP treatment (99.13 ± 20.14 µmol/l, p < 0.0001) and returned to an intermediate level following antimycin exposure (119.7 ± 13.20 µmol/l, p > 0.05, Fig. 2). Cumulus-enclosed oocyte icO_2_ (121.60 ± 13.15 µmol/l, n = 28) reduced significantly following FCCP treatment (98.60 ± 10.21 µmol/l, p < 0.0001) and significantly increased following antimycin exposure (126.00 ± 4.40 µmol/l, p < 0.005, Fig. 2). The presence or absence of cumulus cells did not have any significant effect on the MM2 signal or icO_2_ (p > 0.05).

### icO_2_ throughout in vitro oocyte maturation and embryo development

#### Oocytes

Immature oocyte icO_2_ (94.76 ± 27.28 µmol/l, n = 21) reduced significantly following FCCP treatment (93.39 ± 24.06 µmol/l, p < 0.005) and significantly increased following antimycin exposure (116.6 ± 43.59 µmol/l, p < 0.005, Fig. 3A). Mature oocyte icO_2_ (90.56 ± 18.73 µmol/l, n = 16) was significantly reduced following FCCP treatment (92.97 ± 14.46 µmol/l, p < 0.0005) and increased significantly following antimycin exposure (122.1 ± 29.73 µmol/l, p < 0.005, Fig. 3B).

#### Embryos

Early cleavage stages were grouped into early cleavage (EC) stages (2–7 cell) and late cleavage (LC) stages (8–32 cell) for quantitative analysis. EC basal icO_2_ (84.84 ± 34.37 µmol/l, n = 23) was significantly reduced following FCCP treatment (80.78 ± 34.66 µmol/l, p < 0.0005). icO_2_ increased following antimycin treatment (122.90 ± 28.60 µmol/l, p < 0.0005, Fig. 3C). LC basal icO_2_ (110.1 ± 30.03 µmol/l, n = 26) was similar to FCCP-treated icO_2_ (93.73 ± 36.91 µmol/l, p > 0.05). However, following antimycin treatment (135.70 ± 34.14 µmol/l, Fig. 3D), icO_2_ was significantly higher than basal (p < 0.05) and FCCP treatment (p < 0.0005). EC basal icO_2_ was significantly lower than that of LC embryos (p < 0.05) and blastocysts (p < 0.005).

Blastocyst icO_2_ decreased from basal levels (121.4 ± 16.51 µmol/l, n = 18, Fig. 3E) following FCCP treatment (110.0 ± 20.03 µmol/l, p < 0.0005) and increased with antimycin treatment (151.40 ± 15.16 µmol/l, p < 0.0001).

#### Differences in basal oxygen concentration during oocyte maturation and embryo development

Basal icO_2_ was similar between immature oocytes, mature oocytes, and EC embryos (Fig. 3F). icO_2_ was increased in later stages, with LC embryo icO_2_ significantly higher than that of mature oocytes (p < 0.05) and blastocyst icO_2_ signficiantly higher than that of both mature oocytes (p < 0.005) and LC embryos (p < 0.05).

### Blastocyst ICM and TE visual analysis

Blastocyst images were examined in ImageJ to compare MM2 signal intensity between whole blastocysts, ICM and TE regions (Fig. 4A–C). Mean overall blastocyst basal icO_2_ was 129.23 ± 30.54 µmol/l (n = 82, Fig. 4D). Trophectoderm (TE) oxygen concentration (134.10 ± 32.18 µmol/l) was significantly higher than ICM or overall (p < 0.0001). Inner Cell Mass (ICM) icO_2_ (118.33 ± 29.57 µmol/l) was significantly lower than either TE or overall (p < 0.0001).

### Blastocyst cell allocation ratios

Blastocyst total cell counts (representative images: Fig. 5A–F) varied from 34 to 157 cells and were not normally distributed (n = 31; p = 0.01). Blastocyst cell allocation ratio (% TE) varied from 16 to 88% and were normally distributed (n = 31; p = 0.35). Neither total cell count (75 ± 4.8%; p = 0.07; Fig. 5G), nor cell allocation (57 ± 3.2%; p = 0.52; Fig. 5H) correlated with icO_2_.

### Mitochondrial DNA

Mean MtDNA copy number was 1.75 × 10^5^ ± 1.3 × 10^4^ copies (n = 45). There was no detected correlation between icO_2_ and mtDNA copy number (Fig. 6A; n = 42; R^2^ = 0.05; p = 0.14). mtDNA copy number did not vary significantly between immature oocytes (1.8 × 10^5^ ± 1.6 × 10^4^ copies; n = 10), mature oocytes (1.77 × 10^5^ ± 2.6 × 10^4^ copies; n = 6), early cleavage stage embryos (1.5 × 10^5^ ± 1.8 × 10^4^ copies; n = 19) or blastocyst (2.3 × 10^5^ ± 5.3 × 10^4^ copies; n = 7; p = 0.23, Fig. 6B).

## Discussion

We present a novel method to visualise and quantitatively assess icO_2_ during oocyte maturation and preimplantation embryo development. Labelling with the MM2 probe was user-friendly and reproducible, with quantitation achieved using a simple 2-point calibration curve (Fig. 1). MM2 signal was sensitive to changes in mitochondrial oxygen metabolism and was used to profile icO_2_ at basal, maximal (uncoupled) and non-mitochondrial levels of oxygen consumption using metabolic poisons (Figs. 2, 3). Furthermore, assessment of specific sample regions, namely blastocyst ICM and TE, has been achieved (Fig. 4). MM2 analysis has also been combined with blastocyst cell allocation ratio imaging and qPCR (Figs. 5, 6) and could be used in conjunction with other endpoint analyses.

Basal icO_2_ was similar between immature and mature oocytes (Fig. 3A). The localisation of high MM2 signal intensity, indicating areas of low oxygen concentration, tended to be throughout the ooplasm of the immature oocyte and around the periphery of mature oocytes, comparable to the mitochondrial localisation reported by Sturmey et al.^32^.

To ascertain the effect of cumulus cells on oocyte MM2 loading efficiency and oxygen metabolism, COCs were randomly assigned to overnight culture in IVM media + 5 µg/ml MM2 either with cumulus layers intact or following denudation. Cumulus presence did not affect icO_2_ , with basal, FCCP and antimycin treated values similar between denuded and cumulus enclosed groups (Fig. 2). In both groups, FCCP significantly reduced icO_2_ from basal levels, while antimycin returned oxygen concentration to basal levels. This suggests that (1) cumulus presence did not affect MM2 uptake or sensitivity to changes in oxygen level; and (2) MM2 was sensitive to changes in mitochondrially-regulated oxygen consumption in oocytes. Taken together, this suggests that MM2 is a suitable probe for use in oocytes with or without cumulus.

Oxygen concentration was profiled throughout oocyte maturation and preimplantation development. Basal icO_2_ was highest in blastocysts, at a level significantly higher than in mature oocytes or early cleavage embryos (Fig. 3F). At each stage tested, FCCP treatment did not cause a significant decrease in icO_2_, however antimycin did cause a significant increase in icO_2_ (Fig. 3A–E). Therefore, no significant changes in mitochondrial function during preimplantation development were detected. However, oocyte and embryo mitochondria were sufficiently active at basal level to reduce the icO_2_ by up to 29% in comparison to the minimum represented by antimycin treated icO_2_.

Oxygen consumption is often used as a representative proxy of overall metabolism, and is widely reported to increase progressively throughout preimplantation development^18,51^. By the blastocyst stage, a significant increase in metabolic activity reportedly results in 2–4 times more oxygen being consumed than in cleavage-stage embryos as measured by pyrene fluorescence of small groups of 2–32 embryos^51,52^, extracellular flux analysis of small groups of 6 embryos^25^, or single-embryo nanorespirometry^15^. This increase is most likely due to the requirement for sufficient oxygen to power blastocoel formation. In the present study, blastocyst icO_2_ was higher than that of EC embryos and mature oocytes.

Additionally, further analysis of MM2-labelled blastocysts suggested that the majority of the oxygen was present in the TE, with significantly lower icO_2_ in the ICM (Fig. 4). If icO_2_ was taken purely as an index of oxygen consumption, then this would suggest that (1) blastocysts were less metabolically active than other stages and (2) the ICM was more metabolically active than the TE. However, previous reports have established that this is not the case^17^. In fact, ICM mitochondria tend to be fewer and less active, retaining their globular, immature physiology until post-implantation, while TE mitochondria tend to begin maturation during blastocyst development^17,26^. The present data therefore paints a more complex picture than can be easily explained by reports of oxygen consumption. It is tempting to speculate that oxygen may diffuse rapidly into the TE cells lining the outermost layer of the blastocyst, leading to a higher icO_2_. However rapid oxygen consumption by the highly metabolically active TE mitochondria could leave a reduced amount to diffuse to the ICM. Interestingly, Byatt-Smith et al. predicted that oxygen could diffuse to the centre of smaller embryos (e.g. murine), but cannot diffuse to the centre of the larger bovine embryos^53^. While this is speculative, future study may reveal the dynamics of oxygen diffusion and metabolism in blastocysts. Nevertheless, the present data suggests that the MM2 probe is sensitive to differences in oxygen concentration in different regions of the embryo.

Comparison of blastocyst icO_2_ and total cell count or cell allocation ratio did not reveal any significant correlations (Fig. 5A,B). This data suggests that blastocyst icO_2_ was not significantly dependent on the total number of cells or allocation to TE or ICM. Cell allocation ratios were assayed using the method of Thouas et al.^54^. This method is widely used due to its relative ease and accessibility, and care was taken to expose blastocysts to the PI stain in Triton X for the minimum time to lyse the zona and stain the outermost cells only. However future use of an immunofluorescence approach using antibody markers of the ICM-specific Sox2 and TE-specific Cdx2 could improve specificity and reduce subjectivity^55–57^. Kuno et al. recently reported a positive correlation between chimeric mouse blastocyst OCR measured using a chip-based electrochemical method and increasing total cell count measured by this immunocytochemical approach^23^.

In analyses of oocytes and embryos, the copy number of mitochondrial genomes is often used as an index of the number of individual mitochondria. This is due to reports that, on average, individual oocyte mitochondria each have around 1.3 copies of mtDNA^35,58,59^. In the present study, mtDNA copy number varied greatly within oocyte and embryo developmental stages in the range 0.9–4 × 10^6^ copies (Fig. 6A). This range is comparable to published reports, for example Cotterill et al.^33^ reported 0.74 × 10^6^ copies per ovine MII oocyte, and aligned with a putative 1 × 10^5^ mtDNA copy number threshold for oocyte competence. In the current study no significant differences in mtDNA copy number were observed between oocyte and embryo stages (Fig. 6B). This contrasts to previous reports, in which mtDNA copy number increased significantly during oocyte maturation^60^ and preimplantation embryo development^61^. No correlation was observed between basal icO_2_ and mtDNA count between or within stages (p = 0.14, Fig. 6A,B). This agrees with a recent report from Kuno et al., in which chimeric mouse blastocyst OCR did not correlate with mtDNA copy number^23^. In this study, mtDNA copy number was not correlated to icO_2_ in the samples measured. However, changes in oxidative activity of mitochondria during preimplantation development may also be dependent on changes in mitochondrial maturation and embryo morphology.

This study reports that the commercially available MM2 probe is non-toxic and effective in measuring icO_2_ in mammalian oocytes and preimplantation embryos, including comparison of distinct regions. This method has many potential applications in investigating mitochondrial activity in reproductive tissues, particularly when used in combination with established techniques.

## Materials and methods

All chemicals were obtained from Sigma-Aldrich (Dorset, UK) unless otherwise indicated.

### Bovine tissue acquisition

Ethical approval for animal work was not required for this study as all bovine tissue was derived from non-pregnant animals slaughtered at a local abattoir (John Penny and sons, Leeds, UK) for commercial food production purposes only. The authors were not involved in this process. Bovine reproductive tracts were collected from the abattoir and transported to the laboratory within 2 h of slaughter. The ovaries were dissected from the tracts and live oocytes were aspirated as described below. Bovine embryos were produced in vitro from these oocytes using cryopreserved spermatozoa based on established protocols^51,62,63^. In vitro preimplantation embryo development was maintained to the blastocyst stage for a maximum of 8 days as described below and no animal or embryo transfer studies were conducted.

### In vitro production of bovine embryos

Ovaries were dissected from tracts in the laboratory and washed in pH 7.45 Phosphate Buffer Saline (PBS) comprising 1.058 mM Potassium Phosphate Monobasic, 155.17 mM Sodium Chloride and 2.97 mM Sodium Phosphate Dibasic supplemented with Penicillin, Streptomycin and Amphotericin B (Fungizone) at 39 °C.

Follicular fluid containing live oocytes was aspirated from antral follicles of 2- < 10 mm diameter by aspiration with a pre-warmed syringe fitted with an 18-gauge needle containing a small amount of HEPES-buffered holding medium. Holding medium was Medium-199 supplemented with 4 mM NaHCO_3_, 21.1 mM HEPES, 0.02 mg/ml Heparin, 2 mg/ml Fatty Acid-Free Bovine Serum Albumin (BSA-FAF, A6003) and 100 IU/ml Penicillin/Streptomycin^62^. Cumulus-Oocyte Complexes (COCs) were matured in groups of 50 for 18-24 h in 500 µl *In-Vitro* Maturation (IVM) Medium at 39 °C under humidified 5% CO_2_ in air according to the protocols reported by Hemmings et al.^62^. Serum-free IVM medium consisted of bicarbonate-buffered α-Minimum Essential Medium supplemented with 2 mM glutamine, 0.47 mM pyruvate, 5 µg/ml Sodium Selenite, 10 ng/ml Insulin, 10 ng/ml Long-R3 IGF-1, 0.0006 IU/ml bovine FSH, 0.0003 IU/ml bovine LH, 1 mg/ml BSA-FAF and 100 IU/ml 100 IU/ml Penicillin/Streptomycin.

Oxygen metabolism was quantified in oocytes at 2 discrete stages of meiotic maturity. A subset of cumulus enclosed immature oocytes were analysed immediately following follicle aspiration. Oocytes were denuded of cumulus cells by repeat pipetting through 170 µm and 140 µm EZ-grip embryo handling pipette tips (RI Systems) in 80 IU/ml bovine hyaluronidase in HEPES-buffered MEM at 37 °C. Mature oocytes were collected following 24 h of IVM. Cumulus cells were removed as described above and meiotic progression to MII was confirmed by detection of first polar body extrusion using light microscopy.

In Vitro Fertilisation (IVF) was carried out using frozen-thawed spermatozoa from a bull of proven fertility (Genus, Cheshire, UK). Sperm were centrifuged on a discontinuous Percoll gradient (45:90%) for 30 min at 760 Relative Centrifugal Force (RCF) to select motile sperm. Sperm wash medium was HEPES-Tyrode’s Albumin Lactate Pyruvate (H-TALP) medium comprising 114 mM NaCl, 3.19 mM KCl, 0.45 mM NaH_2_PO_4_, 2 mM NaHCO_3_, 0.26 mM pyruvate, 7.5 mM HEPES, 2.06 mM CaCl_2_, 0.49 mM MgCl_2_, 9.96 mM lactate, 4 mg/ml BSA-FAF and 0.05 mg/ml gentamycin. Selected sperm were washed in H-TALP at 330 × g for a further 5 min before co-incubation with mature COCs in Fertilisation-TALP (F-TALP) at a concentration of 1 × 10^6^ ml^-1^ for 18 h at 39 °C and 5% CO_2_ in humidified air. F-TALP was 114 mM NaCl, 3.19 mM KCl, 0.45 mM NaH_2_PO_4_, 25 mM NaHCO_3_, 0.26 mM pyruvate, 2.06 mM CaCl_2_, 0.49 mM MgCl2, 9.96 mM lactate, 10 µg/ml heparin, 2 µM penicillamine, 1 µM hypotaurine, 4 mg/ml BSA-FAF and 0.05 mg/ml gentamycin.

Putative zygotes were collected from IVF plates in 500 µl HEPES-buffered Synthetic Oviduct Fluid (H-SOF) then denuded of remaining cumulus cells by vortexing for 2 min. H-SOF comprised 111 mM NaCl, 7.16 mM KCl, 1.19 mM NaH_2_PO_4_, 5 mM NaHCO_3_, 0.33 mM pyruvate, 1.5 mM glucose, 33.2 mM lactate, 20 mM HEPES, 1.71 mM CaCl_2_, 4.9 mM MgCl_2_, 9.96 mM lactate, 4 mg/ml BSA-FAF and 100 IU/ml Penicillin/Streptomycin. Putative zygotes were then selected in H-SOF and transferred to SOF culture medium supplemented with amino acids and BSA-FAF (SOFaaBSA) in groups of 40 using a glass pipette. SOFaaBSA comprised 111 mM NaCl, 7.16 mM KCl, 1.19 mM NaH_2_PO_4_, 5 mM NaHCO_3_, 0.33 mM pyruvate, 1.5 mM glucose, 33.2 mM lactate, 0.1 mM glutamine, 1.71 mM CaCl_2_, 4.9 mM MgCl_2_, 9.96 mM lactate, 1 × BME Essential and Non-Essential Amino acids 8 mg/ml BSA-FAF and 100 IU/ml Penicillin/Streptomycin. The zygotes were washed twice in SOFaaBSA before moving to 20 µl pre-equilibrated microdrops under mineral oil for culture in groups of 20 in hydrophobic IVF culture dishes. Embryo culture dishes were incubated at 39 °C under 5% CO_2_, 5% O_2_, 90% N_2_ in humidified air in a Cook MINC mini-incubator for up to 8 days. Mean oocyte maturation rates were 76.2 ± 2.5% and blastocyst development rates were 25.35 ± 9.01%.

### Labelling oocytes and embryos with the MM2 probe

MM2 probe labelling and imaging methods were adapted from those of Kondrashina et al.^38^ and Prill et al.^42^. Pilot experiments confirmed that 24 h incubation in bicarbonate-buffered media led to increased signal intensity in the blue and red channels as well as increased sensitivity in the red channel to changes in oxygen concentration due to mitochondrial inhibition (A). Subsequent experiments showed that extending the incubation time to 72 h had no effect on intensity or signal ratio. Samples were labelled with 5 µg/ml MM2 in 500 µl IVM for oocytes and 5 µg/ml MM2 in 20 µl SOFaaBSA for embryos. Fluorescence values were converted to oxygen concentration in µmol/l using a calibration curve (Fig. 1). Calibration was achieved by plotting signal intensity ratios of fixed oocytes or embryos in 21% oxygen and 0% oxygen environments as described by Prill et al.^42^. Samples were fixed overnight in 2% glutaraldehyde, 2% formaldehyde in PBS to ablate oxygen consuming activity but maintain 3D structure. These samples were imaged in 10mM sodium dithionite in PBS to remove all O2 but otherwise labelled as normal with 5 µg/ml MM2 and imaged as detailed above. Samples for normoxia were fixed to ablate oxygen-consuming activity before rehydration with probe solution to ensure maximal O2 content with zero consumption. Therefore, these were fixed and dehydrated with ethanol before rehydrating with fresh media containing 5 µg/ml MM2 (Fig. 1).

Multiphoton imaging was performed on a Zeiss LSM 710 microscope with Chameleon multiphoton laser. The parfocal distance of the upright multiphoton microscope was insufficient to support use of standard live-cell imaging chambers. Therefore, oocyte and embryo samples were mounted on glass slides, in 5 µl drops of appropriate HEPES-buffered media (e.g. HM for oocytes and HSOF for embryos). Media drops were flanked by 2 layers of labelling tape and overlaid with a glass coverslip. This allowed removal and replacement of the media drop with fresh media supplemented with the required inhibitor for each stage of the experiment.

### Profiling of mitochondrial activity throughout oocyte and embryo development

icO_2_ under basal, maximal and non-mitochondrial conditions were measured sequentially in the same samples as follows. Respiratory chain inhibitors were dissolved in molecular biology grade ethanol, a vehicle which does not significantly change preimplantation embryo oxygen consumption^64^, to investigate how aspects of the bioenergetic profile change throughout preimplantation development. Following measurement of basal oxygen levels, media on the imaging slide was replaced with 5 µl pre-warmed HEPES-buffered media supplemented with 5 µg/ml MM2 and 10 µM Carbonyl Cyanide-P-Trifluoromethoxyphenylhydrazone (FCCP). Samples were incubated for 30 min to allow equilibration of oxygen concentration. This increased OCR to the maximum possible (the maximal respiratory rate). The difference between maximal and basal OCR is the *spare respiratory capacity* and indicative of how close the tissue is to its bioenergetic limit^14,30^. This was followed by treatment with antimycin, an inhibitor of complex III. This effect was immediate, as, once mitochondrial OCR is blocked, oxygen diffuses back into the tissue instantly. Samples were incubated for 5 min to ensure oxygen diffusion had re-equilibrated and for parity between experiments.

### Image analysis

Multiphoton images were examined in ImageJ™. A circular region of interest was selected around each oocyte or embryo sample and mean signal intensity was measured in the blue (reference) and red (oxygen-sensitive) channels. An MM2 probe signal ratio was calculated by dividing red (oxygen-sensitive) signal by total (blue + red) MM2 signal. This was converted to units of oxygen concentration (µmol/l) by referencing to a 2-point calibration curve (Fig. 1C). All subsequent analyses were completed in GraphPad Prism™ 8. Data was analysed for normality by the D’Agostino-Pearson test. Parametric data was analysed by ANOVA with post-hoc Bonferroni test for significant differences between groups, while non-parametric data was tested by Friedman’s test (for paired data) or Kruskal–Wallis test (for unpaired data) with post-hoc Dunn’s test for significant differences between groups. Data comparisons with p < 0.05 were regarded as significantly different.

### Mitochondrial DNA copy number

A subset of samples was flash frozen in liquid nitrogen in 10 µl PBS for mitochondrial DNA (mtDNA) copy number analysis by quantitative real-time Polymerase Chain Reaction (qPCR). A synthesised plasmid was used as a DNA control spike. The method was adapted from those of Cotterill et al.^33^ and Hashimoto et al.^65^. Primers were designed for the mitochondrially-encoded gene COI (cytochrome c oxidase subunit 1) with a PCR product measuring 129 bases. The primer sequences were: 5′ CGTTGTCGCACATTTCCACTA 3′(forward), 5′ GCGAAGTGGATTTTGGCTCAT 3′ (reverse). A spike plasmid (pGEM T-easy vector, Promega, Madison USA) was used as an internal control with forward primer 5′CTAGTGATTGTGCGGGAGAGA3′ and reverse 5′CTTTGAAATTGGCTGGATTGTG3′ and a 152 bp product. The spike was used at a 1/1000 dilution to achieve a similar number of copies in the same order of magnitude as the COI samples through a series of preliminary validation experiments. mtDNA was extracted from a pool of 10 oocytes to identify expected concentrations in these initial validations. Briefly, 10 µl of 2X lysis buffer (2X PCR buffer, 1% Triton X-100 and 200 ng/ml proteinase K) was added to the 10 µl sample for a total sample volume of 20 µl. Cell lysis was performed in a PCR thermal cycler by heating to 55 °C for 30 min then 95 °C 5 min. 2 µl of lysate was used as template in a 25 µl reaction with real-time qPCR master mix comprising: 9.25 µl DNase/RNase free water, (1.25 µl primers (of 10 µM working stocks) and 2.5 µl SYBR Green Master Mix (Applied Biosystems, CA, USA). The qPCR conditions were: Denaturing at 95 °C for 10 min (1 cycle), Amplification at 95 °C for 30 s, 60 °C for 30 s, 72 °C for 1 min (35 cycles), Melt curve at 95 °C, Cooling at 37 °C for 5 min and then maintained at 4 °C until sample collection. qPCR was carried out and data were analysed using a Roche LightCycler 480 and software. The standard curve used to quantify each experimental sample included serial dilutions in the range 10 × 10^3^ to 10 × 10^8^ copies or 0.1–0.000001 ng/ml of the purified PCR product . Mean extraction efficiency calculated as an index of expected vs extracted spike copy number was 82%.

### Cell allocation ratios

Total cell counts and cell allocation ratios were calculated by the method of Thouas et al.^54^. Briefly, blastocysts were first transferred to propidium iodide (100 μg/ml) and 0.001% Triton X-100 in PBS for 30 s to stain TE cells. To prevent labelling of the ICM, blastocysts were immediately washed 3 × in PBS, before transferring to 25 μg/ml Hoechst 3342 in ethanol to label all cell nuclei. Total cell number was counted at 460 nm 3 times and TE cell count was recorded 3 times at 560 nm using a Zeiss Axioscope A1 epifluorescence microscope (Cambridge, UK). The mean of 3 counts were used to calculate the percentage TE out of total cells. The number of ICM cells was calculated by subtracting TE cells from the total Hoechst-stained cells.

### Statistical analyses

Data were tested for normality using the D’Agostino-Pearson test. Parametric data were compared using Student’s t-test or ANOVA with post-hoc Tukey test dependant on the number of groups for comparison. Non-parametric data were compared using Mann–Whitney U test or Kruskal Wallis with post-hoc Dunn’s test. All analyses were performed in GraphPad Prism 6.

The data underlying this article will be shared on reasonable request to the corresponding author.

## Abbreviations

icO_2_: Intracellular oxygen concentration
EGA: Embryonic genome activation
MM2: Pt(II)-5,10,15,20-tetrakis-(2,3,4,5,6-pentafluorophenyl)-porphyrin and poly(9,9-dioctylfluorene) intracellular probe
PtTFPP: Pt(II)-5,10,15,20-tetrakis-(2,3,4,5,6-pentafluorophenyl)-porphyrin
PFO: Poly(9,9-dioctylfluorene)
PBS: Phosphate buffered saline
HEPES: 4-(2-Hydroxyethyl)-1-piperazineethanesulfonic acid
BSA: Bovine serum albumin
BSA-FAF: Bovine serum albumin—fatty acid free
FSH: Follicle stimulating hormone
LH: Luteinising hormone
IVM: In vitro maturation
IVF: In vitro fertilisation
IVP: In vitro production
COC: Cumulus-oocyte complex
MEM: Minimum essential medium
M199: Medium 199
TALP: Tyrode’s albumin lactate pyruvate medium
HM: Holding medium
SOF: Synthetic oviduct fluid
LSM: Laser scanning microscope
FCCP: Carbonyl cyanide-p-trifluoromethoxyphenylhydrazone
mtDNA: Mitochondrial deoxyribonucleic acid
PCR: Polymerase chain reaction
qPCR: Quantitative real-time polymerase chain reaction
COI: Cytochrome c oxidase subunit 1
ICM: Inner cell mass
TE: Trophectoderm
PI: Propidium iodide
ANOVA: Analysis of variance
ATP: Adenosine triphosphate
OXPHOS: Oxidative phosphorylation
AA: Antimycin A

## References

[1] Thouas GA, Trounson AO, Wolvetang EJ, Jones GM (2004). Mitochondrial dysfunction in mouse oocytes results in preimplantation embryo arrest in vitro. Biol. Reprod..

[2] Kasapoğlu I, Seli E (2020). Mitochondrial dysfunction and ovarian aging. Endocrinology.

[3] Nagai S (2006). Correlation of abnormal mitochondrial distribution in mouse oocytes with reduced developmental competence. Tohoku J. Exp. Med..

[4] Wang YM, Qiu MY, Liu Q, Tang H, Gu HF (2021). Critical role of dysfunctional mitochondria and defective mitophagy in autism spectrum disorders. Brain Res. Bull..

[5] Rojas-Charry L, Nardi L, Methner A, Schmeisser MJ (2020). Abnormalities of synaptic mitochondria in autism spectrum disorder and related neurodevelopmental disorders. J. Mol. Med..

[6] Nunnari J, Suomalainen A (2012). Mitochondria: in sickness and in health. Cell.

[7] Beal MF (2000). Energetics in the pathogenesis of neurodegenerative diseases. Trends Neurosci..

[8] Coskun P (2012). A mitochondrial etiology of alzheimer and parkinson disease. Biochim Biophys Acta.

[9] Fragouli E (2015). Altered levels of mitochondrial DNA are associated with female age, aneuploidy, and provide an independent measure of embryonic implantation potential. PLoS Genet..

[10] Berg, J. M., Tymoczko, J. L. & Stryer, L. *Biochemistry* (W H Freeman, 2002).

[11] Leese HJ (2012). Metabolism of the preimplantation embryo: 40 years on. Reproduction.

[12] Leese HJ, Barton AM (1984). Pyruvate and glucose uptake by mouse ova and preimplantation embryos. Reproduction.

[13] Leese HJ (2003). What does an embryo need?. Hum. Fertil..

[14] Birket MJ (2011). A reduction in ATP demand and mitochondrial activity with neural differentiation of human embryonic stem cells. J. Cell Sci..

[15] Lopes AS (2005). Respiration rates of individual bovine in vitro-produced embryos measured with a novel, non-invasive and highly sensitive microsensor system. Reproduction (Cambridge, England).

[16] Trimarchi JR, Liu L, Porterfield DM, Smith PJS, Keefe DL (2000). A non-invasive method for measuring preimplantation embryo physiology. Zygote (Cambridge, England).

[17] Houghton FD (2006). Energy metabolism of the inner cell mass and trophectoderm of the mouse blastocyst. Differ. Res. Biol. Divers..

[18] Lopes AS, Lane M, Thompson JG (2010). Oxygen consumption and ROS production are increased at the time of fertilization and cell cleavage in bovine zygotes. Hum. Reprod..

[19] Shiku H (2001). Oxygen consumption of single bovine embryos probed by scanning electrochemical microscopy. Anal. Chem..

[20] Shiku H (2004). Respiration activity of single bovine embryos entrapped in a cone-shaped microwell monitored by scanning electrochemical microscopy. Anal. Chim. Acta.

[21] Hiramoto K (2017). Development of oxygen consumption analysis with an on-chip electrochemical device and simulation. Anal. Chem..

[22] Kurosawa H (2016). Development of a new clinically applicable device for embryo evaluation which measures embryo oxygen consumption. Hum. Reprod..

[23] Kuno T (2019). A preclinical evaluation towards the clinical application of oxygen consumption measurement by CERMs by a mouse chimera model. Int. J. Mol. Sci..

[24] Obeidat YM (2019). Design of a multi-sensor platform for integrating extracellular acidification rate with multi-metabolite flux measurement for small biological samples. Biosens. Bioelectron..

[25] Muller B (2019). Application of extracellular flux analysis for determining mitochondrial function in mammalian oocytes and early embryos. Sci. Rep..

[26] van Blerkom J (2011). Mitochondrial function in the human oocyte and embryo and their role in developmental competence. Mitochondrion.

[27] Brand MD (2000). Uncoupling to survive? The role of mitochondrial inefficiency in ageing. Exp. Gerontol..

[28] Buttgereit F, Brand MD (1995). A hierarchy of ATP-consuming processes in mammalian cells. Biochem. J..

[29] Lewis AN, Hinrichs K, Leese HJ, Argo CM (2020). Glucose concentration during equine. Reproduction.

[30] Brand MD, Nicholls DG (2011). Assessing mitochondrial dysfunction in cells. Biochem. J..

[31] Zeng HT (2007). Low mitochondrial DNA and ATP contents contribute to the absence of birefringent spindle imaged with PolScope in in vitro matured human oocytes. Hum. Reprod..

[32] Sturmey RG, O’Toole PJ, Leese HJ (2006). Fluorescence resonance energy transfer analysis of mitochondrial:lipid association in the porcine oocyte. Reproduction (Cambridge, England).

[33] Cotterill M (2013). The activity and copy number of mitochondrial DNA in ovine oocytes throughout oogenesis in vivo and during oocyte maturation in vitro. Mol. Hum. Reprod..

[34] Spikings EC, Alderson J, St. John JC, St. John JC (2007). Regulated mitochondrial DNA replication during oocyte maturation is essential for successful porcine embryonic development. Biol. Reprod..

[35] Cummins JM (2002). The role of maternal mitochondria during oogenesis, fertilization and embryogenesis. Reprod. Biomed..

[36] van Blerkom J (2002). Domains of high-polarized and low-polarized mitochondria may occur in mouse and human oocytes and early embryos. Hum. Reprod..

[37] Larsson N-G (1998). Mitochondrial transcription factor A is necessary for mtDNA maintance and embryogenesis in mice. Nat. Genet..

[38] Kondrashina AV (2012). A phosphorescent nanoparticle-based probe for sensing and imaging of (intra)cellular oxygen in multiple detection modalities. Adv. Funct. Mater..

[39] Fercher A, Borisov SM, Zhdanov AV, Klimant I, Papkovsky D (2011). Intracellular O2 sensing probe based on cell-penetrating phosphorescent nanoparticles. ACS Nano.

[40] Dmitriev RI, Zhdanov AV, Jasionek G, Papkovsky D (2012). Assessment of cellular oxygen gradients with a panel of phosphorescent oxygen-sensitive probes. Anal. Chem..

[41] Dmitriev RI, Papkovsky D (2012). Optical probes and techniques for O2 measurement in live cells and tissue. Cell. Mol. Life Sc. CMLS.

[42] Prill S, Andersson A, Papkovsky D, Schmälzlin E (2014). Intracellular O2 measurements: fluorescent microscopy with nanosensors. GIT Lab. J..

[43] Guarino RD (2004). Method for determining oxygen consumption rates of static cultures from microplate measurements of pericellular dissolved oxygen concentration. Biotechnol. Bioeng..

[44] Fraker C (2006). The use of the BD oxygen biosensor system to assess isolated human islets of langerhans: oxygen consumption as a potential measure of islet potency. Cell Transplant..

[45] Lavrentieva A, Majore I, Kasper C, Hass R (2010). Effects of hypoxic culture conditions on umbilical cord-derived human mesenchymal stem cells. Cell Commun. Signal..

[46] Ferreira F (2020). Real-time physiological measurements of oxygen using a non-invasive self-referencing optical fiber microsensor. Nat. Protoc..

[47] Lopes AS, Greve T, Callesen H (2007). Quantification of embryo quality by respirometry. Theriogenology.

[48] Lopes AS (2007). Investigation of respiration of individual bovine embryos produced in vivo and in vitro and correlation with viability following transfer. Hum. Reprod..

[49] Dmitriev RI (2015). Imaging oxygen in neural cell and tissue models by means of anionic cell-permeable phosphorescent nanoparticles. Cell. Mol. Life Sci..

[50] Dmitriev RI, Zhdanov AV, Nolan YM, Papkovsky D (2013). Imaging of neurosphere oxygenation with phosphorescent probes. Biomaterials.

[51] Thompson JG, Partridge RJ, Houghton FD, Cox CI, Leese HJ (1996). Oxygen uptake and carbohydrate metabolism by in vitro derived bovine embryos. Reproduction.

[52] Houghton FD, Thompson JG, Kennedy CJ, Leese HJ (1996). Oxygen consumption and energy metabolism of the early mouse embryo. Mol. Reprod. Dev..

[53] Byatt-Smith JG, Leese HJ, Gosden RG (1991). An investigation by mathematical modelling of whether mouse and human preimplantation embryos in static culture can satisfy their demands for oxygen by diffusion. Hum. Reprod..

[54] Thouas GA, Korfiatis NA, French AJ, Jones GM, Trounson AO (2001). Simplified technique for differential staining of inner cell mass and trophectoderm cells of mouse and bovine blastocysts. Reprod. Biomed. Online.

[55] Liu S (2015). Sox2 is the faithful marker for pluripotency in pig: evidence from embryonic studies. Dev. Dyn..

[56] Yoon JD (2019). GDF8 enhances SOX2 expression and blastocyst total cell number in porcine IVF embryo development. Theriogenology.

[57] Kwak SS, Jeung SH, Biswas D, Jeon YB, Hyun SH (2012). Effects of porcine granulocyte-macrophage colony-stimulating factor on porcine in vitro-fertilized embryos. Theriogenology.

[58] Pikó L, Taylor KD (1987). Amounts of mitochondrial DNA and abundance of some mitochondrial gene transcripts in early mouse embryos. Dev. Biol..

[59] Pikó L, Matsumoto L (1976). Number of mitochondria and some properties of mitochondrial DNA in the mouse egg. Dev. Biol..

[60] Iwata H (2011). Effect of maternal age on mitochondrial DNA copy number, ATP content and IVF outcome of bovine oocytes. Reprod. Fertil. Dev..

[61] McConnell JM, Petrie L (2004). Mitochondrial DNA turnover occurs during preimplantation development and can be modulated by environmental factors. Reprod. Biomed..

[62] Hemmings KE, Leese HJ, Picton HM (2012). Amino acid turnover by bovine oocytes provides an index of oocyte developmental competence in vitro. Biol. Reprod..

[63] Gordon, I. *Laboratory Production of Cattle Embryos*. Biotechnology in agriculture series, vol 27. 2nd ed. CABI (2003).

[64] McKeegan, P. J. *Metabolic Regulation During Early Embryo Development* (2015).

[65] Hashimoto S (2017). Quantitative and qualitative changes of mitochondria in human preimplantation embryos. J. Assist. Reprod. Genetics.

[66] Donnay I, Leese HJ (1999). Embryo metabolism during the expansion of the bovine blastocyst. Mol. Reprod. Dev..

[67] Sturmey RG, Leese HJ (2003). Energy metabolism in pig oocytes and early embryos. Reproduction.

